# Lived Experience and Family Engagement in Mental Health and Substance use Health Research: Case Profiles of Five Studies

**DOI:** 10.1111/hex.70087

**Published:** 2024-11-01

**Authors:** Lisa D. Hawke, Lena Quilty, Branka Agic, Darren B. Courtney, Gray Liddell, Etienne Sibille, Sheila Jennings, Joshua Orson, Holly Harris, Shelby McKee, Cara Sullivan, Sophie Soklaridis, Tarek K. Rajji, Sanjeev Sockalingam

**Affiliations:** ^1^ Centre for Addiction and Mental Health Toronto Canada; ^2^ University of Toronto Toronto Canada

**Keywords:** family members, lived experience, patient engagement, patient‐oriented research

## Abstract

**Introduction:**

People with lived and living experience (PWLLE) and family members (F) can engage in mental health and substance use health research beyond participant roles, as advisors, co‐researchers, equal partners and research leads. However, implementing meaningful and effective engagement is complex.

**Methods:**

This article profiles five research initiatives involving different lived experience engagement structures, situated in a single tertiary care teaching and research hospital.

**Results:**

The profiled projects feature various study designs and stages, ranging from initial priority setting to implementation efforts. The levels of engagement range from consultation to PWLLE/F leadership. Across diverse populations, all embody high‐quality engagement and illustrate that PWLLE/F can have an important impact on a wide range of mental health and substance use health research.

**Conclusions:**

Engagement can be implemented flexibly within a single research institution to meet a wide range of needs and preferences of researchers and PWLLE/F.

**Patient and Public Contribution:**

Each of the research initiatives profiled was conducted with substantial lived experience engagement, as described herein. People with lived and living experience from each research initiative are also included in the authorship team and contributed to this manuscript.

1

Engaging people with lived and living experience (PWLLE) and family members (F) in research is valuable in mental health and substance use health research [1]. Although PWLLE/F have traditionally been research participants, they can be engaged more fulsomely, often as equal partners. Lived experience and family engagement in research aims to make the research more relevant to the priority population [1]. However, meaningful and effective engagement is complex and requires rigorous planning, implementation and evaluation.

A popular typology proposes five levels of stakeholder engagement [2]: (1) researchers ‘inform’ stakeholders; (2) researchers ‘consult’ stakeholders for their feedback; (3) researchers ‘involve’ stakeholders in developing research and taking their perspectives into account; (4) researchers ‘collaborate’ with stakeholders in equal partnerships; and (5) researchers ‘support’ stakeholders in conducting stakeholder‐led research. This typology has the flexibility to be applied to a wide range of projects and structures of engagement.

The Centre for Addiction and Mental Health (CAMH) is a large teaching and research hospital with a substantial PWLLE/F research engagement infrastructure. In addition to a centralized structure, multiple hubs of engagement excellence exist throughout the institution. This includes many project‐ and department‐specific advisory groups and PWLLE/F co‐researchers, advisors and engagement specialists. Together, PWLLE/F support studies across CAMH in alignment with the Strategy for Patient‐Oriented Research (SPOR) [3] and CAMH's strategic direction. A recent scoping review of the engagement literature highlighted the need for more descriptive profiles of engagement in different contexts, using different designs and on different research topics [4]. By adding engagement profiles to the scientific literature, it will be possible to support research teams wishing to develop their engagement practices through examples. This article responds to that evidence gap.

In this article, we present five research projects involving different configurations of lived experience and family engagement, situated in a single tertiary care teaching and research hospital in the mental health and substance use health sector; each project has its own engagement structure and experiences. The cases are summarized in Table 1.

**Table 1:** Summary of the five cases of lived/living experience engagement.

Case	Topic	Description	Engagement structure	Level of engagement	Key contributions (to date)	Challenges
Case 1	Agenda setting	Collaborative exploration of research priorities	Integrated research subcommittee of PWLLE and research members	Collaborate	Establishment of a research agenda for a Recovery College research portfolio	Short timelines for grant submissions, differences in opinion
Case 2	Depression treatment	A bench‐to‐bedside study of theta‐burst stimulation for depression	Integrated knowledge translation	Collaborate	Knowledge translation contributions	Power imbalances
Case 3	COVID‐19 vaccine confidence	A youth‐led photovoice study of youth perspectives	Youth research lead, youth advisory group and lived experience photographer	Support	Youth and lived experience leadership of the study	Youth advisor retention, role delineation
Case 4	Cognitive dysfunction in addiction	Fulsome programme of research	PWLLE advisory group	Involve/collaborate	Co‐development of study design, protocols and communications; multiple novel initiatives and knowledge translation product co‐developed	Advisor retention
Case 5	Integrated care pathway for adolescent depression	Hybrid effectiveness‐implementation, quasi‐experimental, multi‐site, cluster‐controlled clinical trial	Regular contributions from a youth engagement specialist, with occasional youth advisors	Consult/involve	Co‐development of youth‐friendly study operational materials and knowledge translation tools	Navigating schedules and timelines

## 2 Case 1: Co‐Design of a Research Agenda

Recovery Colleges are low‐barrier, strengths‐based, peer‐driven mental health and well‐being‐oriented education programmes that support people in pursuing, maintaining or recovering wellness on their own terms [5]. The Recovery College model is driven by co‐production, by which PWLLE, academics and people with both academic and lived experience collaborate to co‐design and co‐deliver services together [5].

The establishment of a Recovery College at CAMH, known as the Collaborative Learning College, provided the impetus to pursue Recovery College research at the institution. A research subcommittee was established, which is composed of PWLLE, people who access the Recovery College, research professionals with experience in engagement, evaluators and Recovery College staff. The subcommittee meets bi‐weekly to achieve several aims: (1) to co‐develop a Recovery College research agenda; (2) to create a space where people with learned and lived expertise can collaboratively guide Recovery College research; (3) to advise other groups in the mental health community on matters related to Recovery College research and issues of interest to the subcommittee. The work is pursued at the ‘collaborate’ level of stakeholder engagement as described earlier, with equal partnerships among subcommittee members [2].

Although the primary goal of developing a research agenda was predetermined by research team members employed by CAMH, the research subcommittee members collectively decided on the methods and strategies employed to achieve this goal. Members co‐designed the terms of reference, set group expectations and developed a detailed work plan. The work plan included education sessions in areas that members identified as important, collaborating with other committees with cross‐cutting interests, engaging other Recovery College stakeholders to understand their perspectives on research priorities and engaging with Recovery College scholarship. Using the information from this process, committee members drafted a research agenda, identified three overarching research topics and drafted associated research questions to guide Recovery College research at CAMH. Subcommittee members have co‐designed and co‐produced a grant application; short timelines for grant submissions were a challenge, which was mitigated through transparency, dedicated work and a division of tasks. Differences in opinion were resolved by open communication, and occasionally by putting a question to a vote. The subcommittee will continue to collaborate on research outputs such as grant submissions, research projects and knowledge translation activities. Through co‐production, power imbalances are mitigated, leveraging and integrating diverse perspectives in a meaningful way to guide a robust programme of research. The resulting outputs reflect the shared perspectives of the various subcommittee members, including PWLLE.

## 3 Case 2: A Bench‐to‐Bedside Study Applying Integrated Knowledge Translation

A bench‐to‐bedside study aims to optimize the ability of theta‐burst stimulation (TBS), a form of non‐invasive brain stimulation used for the treatment of depression, to enhance brain plasticity in patients with depression. This study features preclinical research, spanning from mouse models to human subjects research.

This study takes an integrated knowledge translation approach to maximize the relevance, impact and usability of this research. As part of the integrated knowledge translation model, knowledge users, including PWLLE/F, are engaged as active participants throughout the project. A PWLLE was involved in the proposal development, and three PWLLE/F are engaged on the project's integrated knowledge translation committee, including one committee family member co‐chair. The committee meets quarterly to guide the project. The study operates at the ‘collaborate’ level of engagement [2].

At the first stage of the study, the PWLLE/F have focused on highlighting the importance of engaging PWLLE/F in preclinical and basic science research. They have thereby helped scientists understand the real‐world relevance of the work they do, closing an important knowledge translation gap. The family co‐chair co‐leads many of the study's knowledge translation activities, fostering shared learning and knowledge exchange. They are also actively contributing to writing a literature review manuscript on integrated knowledge translation in preclinical and basic sciences research. When the study moves into its human‐subjects stage, lived experience and family engagement will support the co‐design of various study procedures. These will notably include the co‐design of recruitment strategies and participant‐facing study materials. PWLLE/F will also continue to be engaged in the dissemination of research findings. A challenge this group encountered was the emergence of potential power imbalances between PWLLE/F and other members of the integrated knowledge translation committee. As a mitigating strategy, the team established a PWLLE/F as the committee co‐chair and co‐created terms of reference document outlining guiding principles, roles and responsibilities. This helped the team move forward equitably, with a shared understanding.

## 4 Case 3: A Youth‐Led Photovoice Study of COVID‐19 Vaccine Confidence

A CAMH research team aimed to understand the perspectives of youth with mental health and/or substance use challenges on COVID‐19 vaccine confidence using a photovoice methodology (i.e., using photography to foster creative expression).

The funding application was written with youth consultation, leading to a tentative study design. Once funded, youth leadership began, following the McCain Model of Youth Engagement [6]. A youth research assistant with lived experience and a bachelor's degree was the ‘youth lead’. She was supported by a youth advisory group and, at the initial stages of the project, two youth engagement specialists. The project was also supported by an adult photographer with lived experience. Although this project was designed at the consultation level of engagement for the funding application stage, it was then conducted at the ‘support’ level, where researchers supported PWLLE in conducting the work [2].

PWLLE refined the methodology and led the study. The youth lead and photographer determined that there would be seven photovoice workshops combining concrete photovoice content and practical exercises. They developed a photography workbook and presentation slides in collaboration with the youth advisory group. The focus group guide was co‐developed as a collaboration between the scientists, the youth lead and the youth advisory group. The workshops and focus groups were facilitated by the youth lead and either the lived experience photographer (workshops) or a young research team member (focus groups). Data were analysed by the youth lead, with support from the scientist and a doctoral trainee who is a PWLLE. Tentative themes, subthemes and representative quotes were brought to the youth advisory group for discussion of the accuracy of the codes and their understanding of the themes; the advisory group provided substantial feedback that led to theme redevelopment and refinement. Two manuscripts were first authored by the youth lead [7, 8]. Additional knowledge translation tools developed by the youth lead and advisory group included a photobook for participants that features their photos, social media sharing, a photo gala, a photography webpage [9] and conference posters. Some challenges were encountered. These included the retention of advisory group members, alongside role definition challenges given the role of a youth research assistant in a lived experience leadership position, which was relatively unique within the engagement infrastructure. The advisor group was refreshed with new membership part way through the study, whereas authentic discussions took place to more clearly delineate the roles of all involved. In sum, the lived experience contribution to the project was substantial, at the leadership level, and was a guiding component of all phases of the study.

## 5 Case 4: A Study of Cognition in Adults Seeking Support for Substance Use Disorder

The Cognitive Dysfunction in the Addictions (CDiA) programme brings together a multidisciplinary team to evaluate the associations between cognition and health in a closely integrated series of studies [10]. This programme includes a longitudinal cohort study (*N* = 400), measures of clinical and cognitive functioning, blood samples, neuroimaging, a qualitative investigation of the lived experience of cognitive difficulties, pilot investigations of neurostimulation and an investigation of wearables.

The research priority was informed by PWLLE in previous research initiatives; upon funding acquisition, a PWLLE/F advisory group was recruited. The advisory consists of eight team members, including six PWLLE and two family members with a range of backgrounds in terms of identity and concurrent mental health and substance use health backgrounds. The studies within this programme of research operate either at the ‘involve’ or ‘collaborate’ level of engagement, depending on the project and its needs [2].

The PWLLE/F advisory group met monthly during study initiation to co‐develop a terms of reference document and a work plan. They also worked to meaningfully shape the programme's research questions, study design and knowledge translation activities. For example, research questions and hypotheses were revised based on PWLLE/F input, as were other core features, such as eligibility criteria, a robust study measure selection process, assessment frequency and procedures related to managing intoxication, withdrawal, crisis and safety. The PWLLE/F advisory group provided numerous key operational details, including study recruitment materials and procedures, communications scripts and visuals and knowledge translation and outreach plans. For example, the PWLLE/F advisory group recommended an increased frequency of follow‐up assessments to maintain participant engagement. They also recommended providing participants with feedback from the measures they complete; both strategies have been received positively by participants. Furthermore, the PWLLE/F advisory group has informed the use of language that is precise and distinguishes between substance use versus substance use disorder and has identified additional opportunities for language to evolve based on study findings (e.g., the use of the term ‘cognitive disabilities’ if appropriate, for alignment with Canadian legal bodies and to promote policy applications). After an intensive programme start‐up phase, meetings moved to quarterly. All new studies associated with the programme meet with the PWLLE/F advisory group before approval, for their guidance and collaboration. Colleagues with lived experience are co‐authors of knowledge dissemination materials and co‐applicants on multiple funding applications under review. PWLLE/F advisory group members have provided key input into future study recruitment, design and knowledge translation plans, which have been received positively by funding bodies. Again, the retention of advisory group members over this long‐term project has been a challenge. New members were recruited after 2 years, but all who have supported the programme are invited to be part of knowledge translation initiatives, according to their interest and availability. Overall, the lived experience contribution to this initiative is not only substantial but also growing, as additional studies and funding applications are developed from this foundational investment.

## 6 Case 5: Implementation of an Integrated Care Pathway for Depression in Adolescents

This research programme is testing the implementation of an integrated care pathway to treat depression in adolescents [11]. The study design is a Type 1 hybrid effectiveness‐implementation, quasi‐experimental, multi‐site, cluster, controlled clinical trial of *N* = 300 youth at six community agencies, with a primary outcome of depressive symptoms [11].

Youth engagement has been a core component of the project since its inception, following the McCain Model of Youth Engagement [6]. Youth have contributed substantially to the development of the care pathway and the study. As part of a full Youth Engagement Initiative, one youth engagement specialist who previously participated in the pathway is a project co‐investigator and is involved on a monthly basis, with more frequent involvement as the need arises. Additional youth provide input as needed in an advisory group that meets three or four times a year. Caregiver feedback is collected via three caregivers consulted in parallel with youth engagement, to improve aspects of the project specifically relevant to caregiver involvement. The study operates at the ‘consult’ or ‘involve’ level of engagement [2], depending on the task at hand.

Youth and caregivers have had a wide range of impacts on the intervention and on the implementation study, from design to knowledge translation. Youth contributions from the previous versions of the intervention and research process were folded into the current trial, providing a youth‐friendly study framework at the outset. Youth directly helped shape the study. For example, they co‐developed recruitment materials and scripts, identified participant compensation processes and trained the research staff on interacting with youth in youth‐friendly ways. They further reviewed the assessment battery and consent forms, co‐developed knowledge translation tools and helped to ensure the suitability of all study components to youth, with attention to youth‐friendliness, accessibility and gender inclusiveness. Youth co‐authored treatment manuals and the published study protocol [11] and led the writing of a process paper describing the youth engagement process [12]. Caregiver engagement has focused on study tasks such as developing psychoeducational materials for caregivers participating in the pathway and troubleshooting low caregiver recruitment into the study. PWLLE/F have had extensive input on the language and examples used in the materials, with the aim of the content being experienced as relevant for the intended audience. PWLLE/F engagement has also helped the study site staff be more engaged in the study, as they are reassured that youth and caregiver perspectives are continually incorporated into the study. One of the biggest challenges encountered during the course of this study has been scheduling meetings with PWLLE/F, who have other priorities in their lives they need to attend to (e.g., school, work and family life). The research team needs to be flexible with the timing of meetings to accommodate those engaged.

## 7 Discussion

This article profiles a variety of research initiatives conducted at a single large academic teaching and research hospital, all employing lived experience engagement. It illustrates the flexibility of engagement approaches to meet the needs of different studies [2]. The profiled projects feature various sub‐populations, study designs, stages of research and models of engagement. Across all projects, PWLLE/F advisors were compensated for their time at a fair, standard hourly rate out of project budgets, reflecting the organization's commitment to meaningful engagement. Despite the variability, all projects show the value of PWLLE/F contributions and illustrate the substantial and positive impacts engagement has had on the research activity at hand.

Across projects, a variety of challenges and mitigating strategies were identified, aligning with the barriers and facilitators to engagement [1]. Some studies experienced attrition or low attendance of PWLLE/F, which was addressed by engaging multiple PWLLE/F to ensure that lived experience voices were always present, and refreshing membership when necessary. Efforts were made to build trusting relationships using friendly check‐ins and check‐outs and by co‐designing group terms of reference documents. Short timelines for grant applications have caused challenges with early engagement, which is a known barrier in the literature; transparency, shared decision‐making, dedicated working meetings and a division of tasks were mitigating strategies. Differences in opinion are a regular challenge frequently experienced in large group meetings, including meetings with PWLLE/F; these are addressed through open, honest discussion and debriefs held frequently and on an ad hoc basis.

A recent literature review has identified that lived experience and family engagement in research has positive impacts on multiple components of the research process [1]. These profiles highlight concrete contributions made by PWLLE/F, which would have led to positive effects across these spheres of impact. The paucity of rigorous research on the impacts of engagement constitutes an evidence gap that researchers are called on to address through lived experience and family‐engaged designs, using a range of qualitative, quantitative and mixed methodologies. Institutional support is a key facilitator of meaningful engagement, as is equitable compensation for their work. A flexible institutional engagement infrastructure made this work possible.

## 8 Conclusions

This article shows that lived experience and family engagement can be implemented in a variety of ways, across study designs, research questions and stages of the research cycle. It shows that PWLLE/F make extensive contributions to research. This includes contributions at early and conceptual levels of developing interventions and establishing priorities. Contributions also include concrete improvements to day‐to‐day research operations supporting study success, such as recruitment and shaping participant‐facing materials. The contributions that PWLLE/F make to integrated and end‐of‐grant knowledge translation are also important, making both scientific and lay knowledge translation outputs relevant to those with lived experience. Notably, by implementing engagement flexibly in manners that meet the needs of each individual project within a single research institution, it is possible to ensure that PWLLE and family voices are amplified throughout the institution's research platform, strengthening the quality of the research conducted. Those designing and implementing engagement initiatives in larger organizations in the mental health and substance use health sector are encouraged to reflect upon this multi‐faceted engagement structure and the experience of these research teams to support their work.

## Author Contributions

**Lisa D. Hawke:** conceptualization, writing–original draft, methodology, project administration, resources, investigation, funding acquisition, writing–review and editing, formal analysis. **Lena Quilty:** conceptualization, writing–review and editing, writing–original draft, investigation, funding acquisition, methodology, formal analysis, project administration, resources. **Branka Agic:** conceptualization, investigation, funding acquisition, writing–review and editing, methodology, formal analysis, project administration, resources. **Darren B. Courtney:** conceptualization, investigation, funding acquisition, writing–review and editing, methodology, resources, formal analysis, project administration. **Gray Liddell:** conceptualization, writing–review and editing, methodology. **Etienne Sibille:** conceptualization, investigation, funding acquisition, writing–review and editing, methodology, formal analysis, project administration, resources. **Sheila Jennings:** methodology, writing–review and editing, conceptualization. **Joshua Orson:** conceptualization, methodology, writing–review and editing. **Holly Harris:** conceptualization, investigation, methodology, writing–original draft. **Shelby McKee:** methodology, writing–review and editing, formal analysis. **Cara Sullivan:** conceptualization, methodology, writing–review and editing. **Sophie Soklaridis:** conceptualization, investigation, funding acquisition, writing–review and editing, methodology, formal analysis, project administration, resources. **Tarek K. Rajji:** conceptualization, investigation, funding acquisition, writing–review and editing, methodology, formal analysis, resources, project administration. **Sanjeev Sockalingam:** conceptualization, investigation, funding acquisition, writing–review and editing, methodology, project administration, resources, formal analysis.

## Ethics Statement

This article does not include original data and was not subject to ethics approval. Each of the studies profiled has its own Research Ethics Board approvals.

## Consent

The authors have nothing to report.

## Conflicts of Interest

The authors declare no conflicts of interest.

## Data Availability

Data sharing is not applicable to this article as no datasets were generated or analysed during the current study.

## References

[1] L. D. Hawke , N. Y. Sheikhan , and T. Rodak , “Lived Experience and Family Engagement in Psychiatry Research: A Scoping Review of Reviews,” Health Expectations 27, no. 3 (2024): e14057. 38678591 10.1111/hex.14057 PMC11056206

[2] G. Bammer, “Stakeholder Engagement Primer: 4. Options for Engagement,” 2021, https://i2insights.org/2021/11/04/options-for-engagement/.

[3] “Strategy for Patient‐Oriented Research: Patient Engagement Framework,” Canadian Institutes of Health Research, 2019, https://cihr-irsc.gc.ca/e/48413.html.

[4] L. D. Hawke , N. Y. Sheikhan , S. Roberts , and S. McKee , “Research Evidence and Implementation Gaps in the Engagement of People With Lived Experience in Mental Health and Substance use Research: A Scoping Review,” Research Involvement and Engagement 9, no. 1 (2023): 32. 37170357 10.1186/s40900-023-00442-5 PMC10176886

[5] S. Soklaridis , H. Harris , R. Shier , et al., “A Balancing Act: Navigating the Nuances of Co‐Production in Mental Health Research,” Research Involvement and Engagement 10, no. 1 (2024): 30. 38454473 10.1186/s40900-024-00561-7 PMC10921621

[6] O. S. Heffernan , T. M. Herzog , J. E. Schiralli , L. D. Hawke , G. Chaim , and J. L. Henderson , “Implementation of a Youth‐Adult Partnership Model in Youth Mental Health Systems Research: Challenges and Successes,” Health Expectations 20, no. 6 (2017): 1183–1188. 28295940 10.1111/hex.12554 PMC5689223

[7] S. Mckee , T. Halsall , N. Y. Sheikhan , R. Knight , J. Henderson , and L. D. Hawke , “‘Pictures Helped Me Understand It in a way Words Couldn't’: Youth Reflections Participating in a Youth‐Led Photovoice Study,” PLoS One 19, no. 9 (2024): e0308165. 39240902 10.1371/journal.pone.0308165 PMC11379270

[8] S. McKee , N. Y. Sheikhan , S. Patenaude , et al., “‘Is It Safe? Is It Not?’ A Youth‐Led Photovoice Study of Youth Perspectives of COVID‐19 Vaccine Confidence,” Health Expectations 27, no. 5 (2024): e70051. 39369276 10.1111/hex.70051 PMC11456145

[9] “Photovoice Project: Youth Perspectives on COVID‐19 Vaccine,” Centre for Addiction and Mental Health, 2024, https://www.camh.ca/en/science-and-research/research-connect/youth-perspective-on-covid-19-vaccine/photovoice-project.

[10] Y. S. Nikolova , A. C. Ruocco , D. Felsky , et al., “Cognitive Dysfunction in the Addictions (CDiA): A Neuron to Neighbourhood Collaborative Research Program on Executive Dysfunction and Functional Outcomes in Outpatients Seeking Treatment for Addiction,” medRxiv, Published ahead of print, August 31, 2024, 10.1101/2024.08.30.24312806.

[11] D. B. Courtney , M. Barwick , B. Amani , et al., “An Integrated Care Pathway for Depression in Adolescents: Protocol for a Type 1 Hybrid Effectiveness‐Implementation, Non‐Randomized, Cluster Controlled Trial,” BMC Psychiatry 24, no. 1 (2024): 193. 38459453 10.1186/s12888-023-05297-4 PMC10921633

[12] M. Prebeg , J. Relihan , K. Darnay , et al., “Youth Partner Engagement in the Development of an Integrated Care Pathway for the Treatment of Adolescents With Depression,” PsyArXiv, Published ahead of print, July 07, 2023, 10.31234/osf.io/kyzcj. PMC12435350 40958898

